# A phase II clinical trial to assess the safety of clonidine in acute organophosphorus pesticide poisoning

**DOI:** 10.1186/1745-6215-10-73

**Published:** 2009-08-20

**Authors:** Polwattage MS Perera, Shaluka F Jayamanna, Raja Hettiarachchi, Chandana Abeysinghe, Harindra Karunatilake, Andrew H Dawson, Nick A Buckley

**Affiliations:** 1 South Asian Clinical Toxicology Research Collaboration, Faculty of Medicine, University of Peradeniya, Sri Lanka; 2 Australian National University Medical School, Canberra, Australia; 3 Prince of Wales Hospital Clinical School, University of NSW, Australia; 4 Polonnaruwa General Hospital, Sri Lanka; 5 Chilaw General Hospital, Sri Lanka

## Abstract

**Background:**

An estimated 2–3 million people are acutely poisoned by organophosphorus pesticides each year, mostly in the developing world. There is a pressing need for new affordable antidotes and clonidine has been shown to be effective in animal studies. Our aim was to determine the safety of clonidine given as an antidote in adult patients presenting with signs or symptoms of acute organophosphate ingestion.

**Methods:**

This study was a dose finding, open-label, multicentre, phase II trial. Forty eight patients with acute organophosphate poisoning were randomized to receive either clonidine or placebo: Four to receive placebo and twelve to receive clonidine at each dose level. The first dose level was an initial loading dose of 0.15 mg followed by an infusion of 0.5 mg of clonidine over 24 hours. The initial loading dose was increased to 0.3 mg, 0.45 and 0.6 mg. at all dosing levels however the subsequent infusion remained at 0.5 mg of clonidine over 24 hours.

**Results:**

The baseline characteristics of both groups were similar. The trial was stopped after completion of the 3^rd ^dosing level. At the 1^st ^and 2^nd ^dosing level there were no reported adverse drug reactions. At the 3^rd ^dosing level 5 patients (42%) developed significant hypotension during clonidine treatment that responded to intravenous fluids. There were no statistical differences in ventilation rate, pre and post GCS, and mortality rates over all levels.

**Conclusion:**

Our findings suggest use of moderate doses of clonidine in acute organophosphate poisoning can be used without causing frequent clinical problems but that higher doses are associated with a high incidence of hypotension requiring intervention. Further studies are needed to study the efficacy of clonidine as an antidote in organophosphate poisoning.

**Trial registration:**

Current Controlled Trial ISRCTN89917816.

## Background

Each year at least 300,000 thousand people die from deliberate ingestion of pesticides [1]. These deaths are responsible for about a third of the global burden of illness from suicide [2]. In many countries this is the leading cause of lives lost in early to middle adult life. Anti-cholinesterase pesticides account for two thirds of these pesticide poisoning deaths and these are most common in rural areas of the developing world where the World Health Organization (WHO) estimates 2–3 million people are affected annually. In Asia alone 200,000 deaths per year result from intentional ingestion of organophosphorus (OP) compounds; poisoning has an estimated case fatality of between 5 and 30% [3]. Poisoning in Sri Lanka is always among the five leading causes of mortality and morbidity and accounts for about 80,000 hospitalizations and 3000 deaths per year [4]. This problem is compounded by the fact that approximately 35% of patients acutely poisoned with organophosphates require intensive care and mechanical ventilation [5]. This is despite conventional antidote treatment with atropine and pralidoxime [3]. One reason for this high mortality is the failure of pralidoxime to be effective in many clinical situations. The major mechanism of toxicity from organophosphorus pesticides is inhibition of the synaptic acetylcholinesterase enzyme. Acetylcholinesterase inhibition is initially reversible but eventually becomes irreversible, a process which is commonly termed "ageing". Acetylcholinesterase inhibition leads to excessive accumulation of acetylcholine at nicotinic and muscarinic synapses leading to widespread clinical effects culminating in neuromuscular and respiratory failure.

Acute respiratory failure leading to death is a major clinical problem in OP poisoning. In one study involving 376 patients intoxicated with OP, 90 (24%) required intubation and ventilatory support during their hospital admission. Forty-six (51%) of the intubated patients died[6]. Acute respiratory failure produced by cholinesterase inhibitors is mainly due to inhibition of central respiratory drive and direct pulmonary toxicity [7]. Atropine is effective at competitively blocking the effects of acetylcholine at the muscarinic receptor. Oximes, such as pralidoxime, can reactivate acetylcholinesterase but the ability to reverse acetylcholinesterase (AChE) inhibition with oximes varies with the type of pesticide ingested and time to treatment as both these factors affect the rate of enzyme ageing [8,9]. Moreover, as pralidoxime has poor CNS penetration its effects are largely restricted to the peripheral nervous system. In Sri Lanka and much of rural Asia, delays in initiating treatment and the use of organophosphates that are associated with rapid ageing of acetylcholinesterase inhibition contribute significantly to treatment failure with pralidoxime. This treatment failure also contributes to a high treatment cost as pralidoxime is relatively expensive [10].

In this clinical setting it is appropriate to examine inexpensive antidotes operating through synergistic mechanisms that are not dependent upon acetylcholinesterase reactivation. One approach is to reduce the amount of acetylcholine released into the synapse.

Clonidine, a centrally acting antihypertensive agent, is known to decrease the presynaptic synthesis and release of acetylcholine. This pre-synaptic effect is greater in the central nervous system than in peripheral cholinergic synapses [11]. Administration of clonidine to animals poisoned with organophosphorus pesticides has been shown to improve outcome in numerous studies [12-16]. Other studies found that both atropine and clonidine in OP-intoxicated mice showed increased survival time with delay in development of whole body tremor and loss of righting reflex [17] and reduced salivation [18]. Doses of 0.3 and 1.0 mg/kg were both effective in reducing mortality from soman (1/7 deaths vs 14/16 in controls) [14]. In a study that explored a wider range of doses (between 0.125 and 8 mg/kg IM) in rats poisoned with soman, the optimal doses in reducing mortality were 0.5 and 1.0 mg/kg. Adverse effects were markedly more obvious at doses greater than 0.5 mg/kg but minor at that dose and below [16]. A dose of 0.5 mg/kg was highly effective in soman poisoning but a dose of 0.2 mg/kg when combined with a sub-therapeutic dose of atropine was as effective as 0.5 mg/kg [19]. This suggests an additive or synergistic effect of clonidine with atropine. Lower doses may therefore have the optimal balance of risks and benefits in clinical practice [12].

Clonidine was shown to be ineffective against echothiopate, an organophosphorus pesticide that does not enter or have effects on the central nervous system. Therefore, it is likely the antidotal effect of clonidine is predominately centrally mediated [14]. This may indicate there will be further clinical benefit over current antidotes as OP-induced centrally mediated respiratory failure is unlikely to respond to oximes and is likely to involve non-muscarinic receptors that are not blocked by atropine. Clonidine may also inhibit the release of inflammatory neuropeptides that may be involved in pulmonary toxicity [20].

Clonidine has been used widely in humans for decades. Most commonly, it has been used for the treatment of high blood pressure (particularly in pregnancy), opioid withdrawal, and sedation in intensive care. The pharmacological mechanism involves blockade of central alpha-2-adrenergic receptors. The most frequent adverse effects are low blood pressure and sedation, but these effects are generally not serious or life-threatening. The typical doses used in narcotic withdrawal and hypertension are between 0.15 mg to 0.3 mg four times daily. Higher doses are used intravenously for sedation: – a bolus of 0.0015 to 0.005 mg/kg (up to a maximum of 0.6 mg) and then an infusion of 0.0003 mg/kg/h [21]. These routinely used sedating intravenous doses informed the choice of doses explored within this trial.

Our aim was to explore the effect of a range of doses of clonidine, primarily to look at which doses were well tolerated in patients with OP poisoning.

## Methods

### Trial Design and Study Population

**Figure 1:**
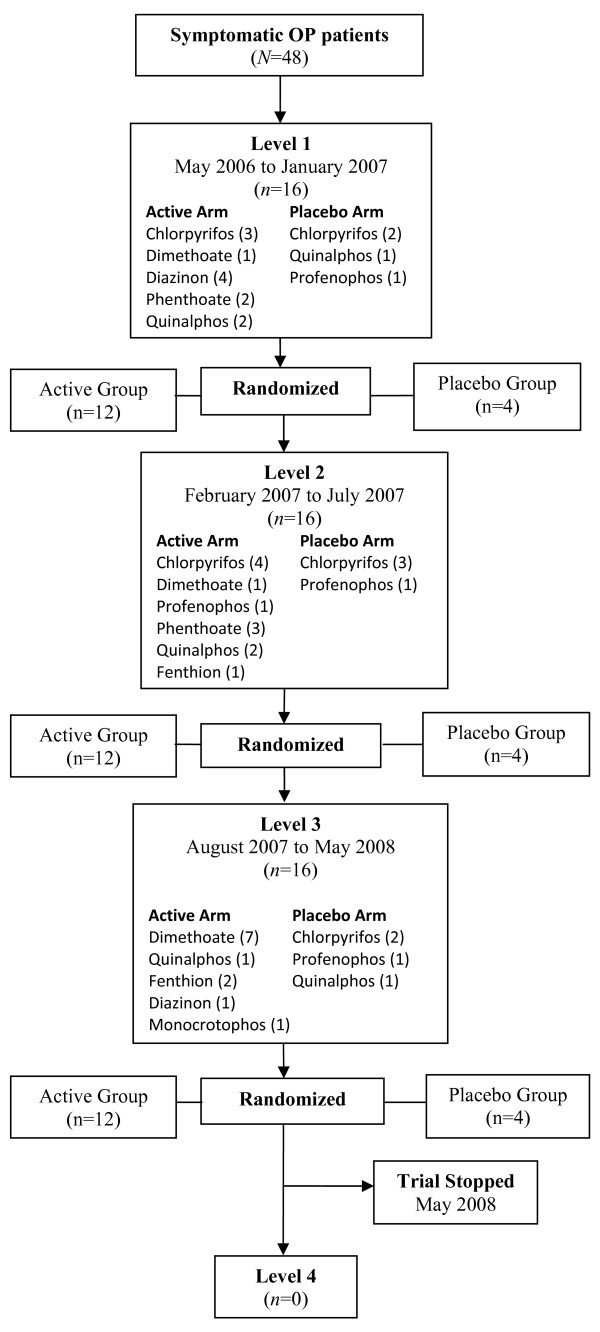
**Trial flowchart**.

The study was planned as an open-label Phase II progressive dose-ranging study with four doses explored in stages. Sequential groups of 16 patients were randomized to receive either a bolus dose of clonidine in a stage-wise manner (0.15, 0.30, 0.45, or 0.6 mg) and then a constant 0.5 mg clonidine/24 hours or to receive a glucose infusion at every level (Figure 1). After written informed consent was obtained, randomization was performed centrally by a computer-generated randomization program that randomly assigned subjects within each dose cohort to active-drug or placebo treatment in a 3:1 ratio. Thus, at each treatment level, twelve subjects were to receive clonidine and four were controls. Although the patients were blinded, the treating physician, nurses and clinical research assistants were aware of the treatment allocation.

The patients were recruited between May 2006 and May 2008 at the Chilaw and Polonnaruwa General hospitals in rural Sri Lanka. Subjects were recruited who had symptomatic acute OP poisoning. Eligibility required a history of OP pesticide ingestion and cholinergic features and/or prior administration of atropine for such features. Where possible, the specific type of OP pesticide ingested was confirmed by identification of the brand name or examination of the bottle. Pregnant patients and those younger than 16 years of age were excluded. Subjects were also excluded if they were hypotensive (BP <90/50 mmHg) on presentation, if they ingested other substances in addition to OP pesticide, or if they had other serious medical conditions (e.g. cardiovascular disease, renal or hepatic failure). Other than study medication, patients received standard medical care under the hospital's admitting consultant physician. This usually included titrated doses of atropine and pralidoxime chloride 1 gram 6^th ^hourly for 48 hours.

The study received ethical approval from the Sri Lanka Medical Association (ERC/05-008), and the Australian National University Human Research Ethics Committees (2005/159). It was registered with the International Trial Registry Number ISRCTN89917816.

### Dosing Regimen

Catapress^® ^– clonidine 150 ug/ml batch number 526088c produced by Boehringer Ingelheim, New South Wales, Australia was used. The initial loading dose (0.15 mg, 0.3 mg, or 0.45 mg) was given over 10 minutes diluted to 10 ml normal saline. At all dose levels an infusion of 0.5 mg of clonidine was then diluted in 500 ml of 5% dextrose and given over 24 hours with infusion pumps. 'Placebo' loading doses and infusions were an equal volume of 5% dextrose. Drugs were prepared shortly before use by a registered pharmacist.

### Assessment of Outcomes

All patients were prospectively monitored with regular measurement of their pulse, BP and saturation via a bedside vital signs monitor by medically qualified and trained research assistants. They also recorded patient demographics, the type and amount of organophosphate, alcohol co-ingestion and other co-morbid conditions and recorded the occurrence of specific clinical outcomes: death, ventilation, hypotension (BP <80/40), and adverse reactions to clonidine. The doses of atropine prior to, during and after clonidine treatment were also recorded.

The trial was an exploratory dose-finding phase II study to guide safe dosing in subsequent larger studies. Therefore it had a low power to detect differences in major clinical outcomes and any therapeutic efficacy. No formal sample size or power calculation was performed. The specified primary outcome measure was the proportion of patients in each of the randomized group levels who either were ventilated or died. Secondary outcome measures included decreased level of consciousness and hypotension defined by a manual blood pressure reading of equal to or less than 80/40 mmHg. The change in heart rate and blood pressure and the requirements for atropine over the 24 hours of clonidine of treatment were also compared.

### Data Analysis

Analysis of major clinical outcomes was done on all randomized patients based upon intention to treat. All patients who were randomised were included in the analysis. As management protocols did not change over the course of the study patients randomised to placebo at each dose level were aggregated into one group to facilitate an examination for adverse effects.

Statistical analysis was performed using STATA v8 and Prism V5. Clinical characteristics were summarized using counts (percentages) for categorical data and median (interquartile range [IQR]) for non-normally distributed continuous variables. The data on age, amount of poison ingested and atropine dosing were analyzed using the Kruskal Wallis test. Thereafter Dunn's test was used whenever an overall difference was noted. Proportions were compared with the Chi square test. Two-way repeated measures ANOVA and whenever appropriate Bonferroni post hoc pair-wise comparison was done to compare blood pressure readings over the entire 24 hours.

## Results

Between May 2006 and May 2008 we recruited 48 symptomatic acute OP pesticide poisoning cases from two peripheral hospitals in rural Sri Lanka. One patient died just after randomization prior to clonidine treatment while thirty-five received the initial doses of the active drug (0.15 mg, 0.3 mg or 0.45 mg) and 12 study subjects received a placebo. At the 3^rd ^dosing level, low blood pressure readings (<80/40) were recorded in 5/11 patients during infusions; therefore, the trial was stopped before proceeding to the cohort at the highest planned dose (0.6 mg bolus).

The baseline characteristics and the time to start placebo or treatment were reasonably balanced across all groups (table 1). It is worth noting that more patients in the placebo arm had ingested chlorpyrifos (7; 58%), while in the 3^rd ^dosing level there were a much higher number of dimethoate ingestions (7; 58%). This related to a change in the most common OP ingested (Figure 1). There were three deaths; two of these were dimethoate poisonings while the other had ingested quinalphos. All developed hypotension and respiratory failure requiring ventilator support prior to death.

**Table 1:** Baseline Characteristics according to randomized treatment with clonidine.

	Dosing Level				
	Placebo	Level 1	Level 2	Level 3	P Value
	n = 12	n = 12	n = 12	n = 12†	
Males	10 (83%)	11 (91%)	10 (83%)	9 (75%)	0.75
Age (years)*	32 (23–47)	31 (26–38)	26 (22–34)	37 (31–46)	0.2
Amount of poison ingested (mL)*	60 (50–100)	55 (50–100)	50 (30–80)	80 (35–100)	0.64
Time to admission after ingestion (hrs)*	3.5 (3–6)	5(1–7)	4 (3–7)	3 (3–3)	0.58
Time to start clonidine from ingestion (hrs)*	6 (3–7)	6 (4–9)	6(5–8)	5 (4–5)	0.41
Direct admission	4 (33%)	5 (42%)	4 (33%)	5 (42%)	0.95
Alcohol ingestion	5(42%)	2(17%)	4(33%)	6(50%)	0.28
Co-Morbid Illness^€^	0 (0%)	2(17%)	1(8%)	2(17%)	0.59
Pralidoxime used	4 (33%)	7 (58%)	5 (42%)	2 (17%)	0.25
Pesticide:					
Chlorpyrifos	7 (58%)	3(25%)	4 (33%)	0 (0%)	*0.018*^£^
Dimethoate	0 (0%)	1 (8%)	1 (8%)	7 (58%)	*0.001*^¥^
Others^≠^	5 (42%)	8 (67%)	7(58%)	5 (42%)	

Data are number (%) unless otherwise indicated. *Median (IQR).). †One patient died after randomization before receiving clonidine. Comparisons between the three groups were made using a Kruskal-Wallis. ^£^P < 0.05 by Kruskal-Wallis with post hoc Dunn's test between level 1 and placebo. ^¥^P < 0.05 by Kruskal-Wallis with post hoc Dunn's test between level 3 and placebo. ^€^Co-morbid illness included cardiac, pulmonary, psychiatric, neurologic and undiagnosed disorders. ^≠^Diazinon, Phenthoate, Profenophos, Monocrotophos, Fenthion, Quinalphos.

The atropine doses were similar with respect to bolus doses and there was a statistically significant difference between groups in the infusion rates due to higher pre-infusion atropine doses in the 3^rd ^vs. 2^nd ^dosing levels (Dunn's test, P < 0.05) (Table 2). The total atropine duration was similar across all groups.

**Table 2:** Atropine Doses in RCT pre and post commencement of the clonidine infusion.

	Placebo	Level 1	Level 2	Level 3	P value
	n = 12	n = 12	n = 12	n = 12†	
*Pre clonidine dosing*					
Atropine Bolus (mg)	9 (2–28)	6 (1–12)	2 (1–6)	6 (3–24)	0.19
Atropine Infusion (mg/hour)	10 (1–24)	1 (1–1)	1 (1-1)	5 (1–11)	*0.008*^£^
*Post clonidine dosing*					
Atropine Bolus (mg)	6 (2–9)	1 (0–1)	0(0–3)	1 (1–15)	0.05
Atropine Infusion (mg/hour)	2 (0–16)	1 (1-1)	1 (0–2)	6 (1–15)	0.47

Data are Median (IQR); †One patient died after randomization before receiving clonidine. Comparisons between the three groups were made using a Kruskal-Wallis.^£^P < 0.05 by Kruskal-Wallis with post hoc Dunn's test between level 2 and level 3 groups.

There was no statistical difference in primary clinical outcomes analyzed based on intention to treat (i.e. included the patient who died prior to receiving clonidine) (Table 3). However, at the third (highest) dosing level, 4 (33%) patients were ventilated and 3 deaths occurred, all in ventilated patients. One patient (9%) developed a single episode of sinus pauses less than 3 seconds in duration 6 hours after commencing the infusion. The infusion was stopped thereafter in this patient.

**Table 3:** Clinical outcomes according to randomized allocation of clonidine treatment (ITT).

	Placebo	Level 1	Level 2	Level 3	P value
	n = 12	n = 12	n = 12	n = 12†	
GCS < 15					
Pre Intervention	7(58%) [30 to 83]	4(33%) [12 to 62]	7(58%) [30 to 83]	5(41%) [17 to 70]	0.52
Post Intervention	5(42%) [17 to 70]	3(25%) [7 to 54]	5(42%) [17 to 70]	6(50%) [23 to 77]	0.64
GCS ≤ 8					
Pre Intervention	2(17%) [3 to 45]	0(0%) [0 to 22]	1(8%) [0.4 to 35]	1(8%) [0.4 to 35]	0.53
Post Intervention	1(8%) [0.4 to 35]	1(8%) [0.4 to 35]	1(8%) [0.4 to 35]	2(17%) [3 to 45]	0.88
Hypotension*	0(0%) [0 to 22]	0(0%) [0 to 22]	0(0%) [0 to 22]	6(50%) [23 to 77]	*<0.001*^£^
Ventilation	4(33%) [12 to 62]	3(25%) [7 to 54]	0(0%) [0 to 22]	4(33%) [12 to 62]	0.17
Death	1(8%) [0.4 to 35]	0(0%) [0 to 22]	0(0%) [0 to 22]	3(25%) [7 to 54]	*0.08*
Ventilated or death	4(33%) [12 to 62]	3(25%) [7 to 54]	0(0%) [0 to 22]	4(33%) [12 to 62]	0.17

Data are in n (%) [95% CI];*Blood pressure less than 80/40 mmHg. †One patient died after randomization before receiving clonidine. ^£^P < 0.05 by Kruskal-Wallis with post hoc Dunn's test between level 3 and placebo.

The third dosing level also had a statistically higher (p < 0.001) incidence of hypotension; five (45%) of the 11 patients who received clonidine developed hypotension (BP <80/40) at some stage. The clonidine in these 5 patients was temporarily discontinued at this time. In these patients the lowest recorded blood pressure was 71/53 mmHg. All five had some decrease in systolic, diastolic and mean arterial pressure within the first hour of infusion but lowest values were delayed toward the middle of the infusion. They all returned to normal range with intravenous fluids (20 ml/kg rapid bolus) within approximately 60 minutes without any sequelae.

**Figure 2:**
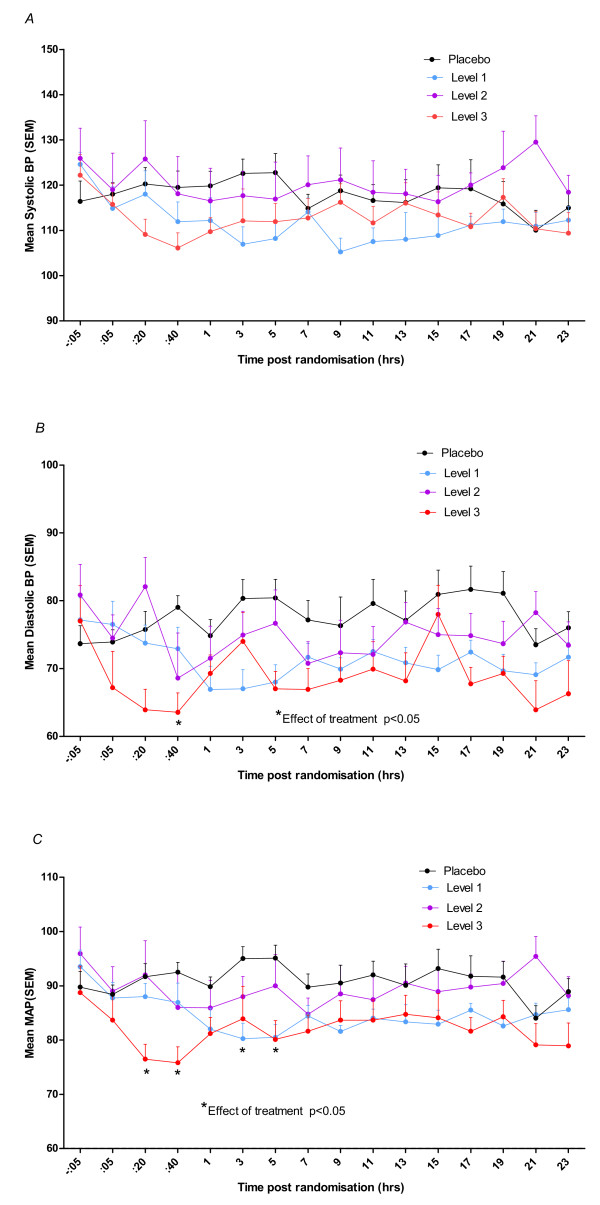
**Time course of (A) systolic, (B) diastolic and (C) mean arterial pressure (MAP) changes in placebo group (n = 12), level 1 (n = 12), level 2 (n = 12) and level 3 (n = 11) treatment groups in 24 hours**. Note that in five patients at level 3, the clonidine was stopped at some stage because of hypotension (BP <80/40 mmHg) and patients were given extra fluids. Two-way ANOVA showed significant differences between clonidine doses in diastolic blood pressure (effect of treatment: *F *= 5.40, df = 3, *P *< 0.05; time post randomization: *F *= 1.31, df = 15, *P *> 0.05) and mean arterial pressure (effect of treatment: *F *= 5.62, df = 3, *P *< 0.05; time post randomization: *F *= 1.01, df = 15, *P *> 0.05) but not in the systolic blood pressure (effect of treatment: *F *= 2.46, df = 3, *P *> 0.05; time post randomization: *F *= 1.02, df = 15, *P *> 0.05). The Bonferroni post-test was also significant at the asterixed data points vs placebo (P < 0.05).

In contrast, the lower two doses were well tolerated, although statistically significant effects on blood pressure were noted (Figure 2). The mortality and need for ventilation was (non-significantly) lower than in the placebo group, as were atropine doses. No important adverse drug events were recorded.

There was a statistically significant higher rate of mean observed blood pressure recordings in the active groups (8.3 ± 2.6 vs 4.9 ± 1.4; p < 0.001).

Figure 2 shows the pattern of systolic, diastolic and mean arterial pressures in all cohorts. There was no statistically significant effect of treatment on systolic blood pressure of the treatment group when compared to the placebo arm (two-way repeated measures ANOVA with Bonferroni posttest). The second dosing level showed a statistically significant lower mean arterial pressure which occurred 3 and 5 hours (p < 0.05) into the infusion. The third dosing level showed statistically significant lower diastolic pressure at 40 minutes and a significantly lower mean arterial pressure at 20 & 40 minutes and at 5 hours post the loading dose during the infusion (p < 0.05). The maximum decrease from baseline in systolic blood pressure, diastolic pressure and mean arterial pressures ranged from 30 to 57 mmHg, 21 to 40 mmHg and 23 to 38 mmHg respectively across all clonidine doses (Table 4).

**Table 4:** Effect of treatment on the maximum change in systolic, diastolic and mean arterial blood pressure.

Treatment arm	Placebo (n = 12)	Level 1 (n = 12)	Level 2 (n = 12)	Level 3 (n = 11)
	Systolic blood pressure			
Baseline mean (SD)	116(16)	125(9)	123(26)	122(15)
Maximum change	-36	-30	-57	-32
Minimum change	0	0	1	1
	Diastolic blood pressure			
Baseline mean (SD)	75(9)	77(11)	80(16)	76(17)
Maximum change	-21	-24	-28	-40
Minimum change	1	1	0	0
	Mean arterial pressure			
Baseline mean (SD)	88(10)	93(9)	95(18)	91(16)
Maximum change	-25	-23	-35	-38
Minimum change	0	0	0	0

## Discussion

Clonidine has been studied extensively in the past and has been used widely in humans for decades. The pharmacological mechanism involves blockade of central alpha-2-adrenergic receptors and imidazoline receptors [22]. The most frequent adverse effects are low blood pressure and sedation.

Hypotension was the most frequently observed event. There were small decreases in mean blood pressure at all doses. At the third dosing level the hypotension required treatment with fluid boluses and temporary stoppage of the infusion; the blood pressure thereafter returned to normal after 15 to 30 minutes. None of these patients required vasopressor support; however, the investigators decided that there was a low likelihood that higher doses would be well accepted by the treating staff and the trial was ceased at this time. A dose in the order of 0.15 or 0.3 mg clonidine bolus and 0.5 mg/24 h infusion appears most likely to be clinically acceptable for further studies in this low-resource setting.

The episodes of hypotension may simply be explained by the larger doses of clonidine, but other factors may have contributed. The acute effects of organophosphorus pesticide include hypotension. This is much more common with dimethoate poisoning, which also has a higher case-fatality (23%) than chlorpyrifos (8%) or fenthion (16%)[8]. Seven (58%) of the 12 patients who had received the highest dose had ingested dimethoate; 5 of these were either unconsciousness, hypotensive and/or required intensive care treatment and this may account for the higher death rate (3/12;25%) at this level. Other aggravating factors, such as gastrointestinal decontamination procedures or persistent vomiting and diarrhea, may also lead to volume depletion. This highest dose group also had the lowest use of pralidoxime. Pralidoxime is known to increase blood pressure and this may also have contributed to lower blood pressures in this group.

The sedative effects of clonidine are attributed to activation of the alpha-2-adrenergic receptor in the locus coeruleus which suppresses the spontaneous firing rate of the nucleus and increases the activity of GABA[23]. Sedation was not pronounced in our patients. Clonidine in the setting of OP poisoning may possibly be less sedating as acetylcholine is a CNS stimulant.

Other than hypotension no other important adverse drug events occurred. One patient in the high clonidine dose group had a brief (less than 3 sec) sinus pause recorded which might have been related to clonidine. Although dysrhythmia associated with clonidine is rare[22], there have been a few case reports of AV block and cardiac dysrhythmia [24,25]; these occurred in conjunction with other substances also reported to cause such effects making causality difficult to establish. Similarly, for our patient, dysrhythmia have been observed with OP poisoning alone.

We found no significant difference in the primary outcome measure of 'ventilation or death' all of which occurred post-treatment. This was not surprising as although this was our pre-specified primary outcome, our trial was inadequately powered to detect anything but an extreme difference in this outcome. While not statistically significant, the first two doses were well tolerated and associated with better than average outcomes; the case-fatality within the trial (8%) was lower than the overall case fatality of 20% [26] for acute symptomatic organophosphorus pesticide poisoning.

Our study had three main limitations. The most important is that it was small and designed to guide future studies rather than powered to reach definitive conclusions. It was also an open-label trial. This was required to address concerns on the hypotensive effect of clonidine raised by the attending physicians and ward staff. Thus, knowledge of treatment allocation by the treating physician might have influenced monitoring and treatment decisions. For example, blood pressure was measured twice as frequently in the active arms as compared to the placebo arm. This may have lead to a bias towards more frequent detection of low blood pressure in the treatment arms. Third, there was not random allocation between different doses (only between placebo and a particular dose). This led to substantive baseline imbalances between clonidine dose levels in the OP taken, probably due to seasonal variation in patterns of pesticide use (Figure 1). The greater number of severely unwell patients into the highest dose cohort may also be due to the ward staff being more confident in recruiting such patients as the trial progressed.

Thus it is inappropriate to interpret the results of this trial in isolation. Its primary purpose was to determine if larger more definitive trials can be ethically justified. The secondary purpose was to provide data relevant to the design of further trials.

## Conclusion

In our limited pilot study clonidine was well tolerated at the two lowest dosing levels suggesting that moderate doses of clonidine (150 μg or 300 μg bolus and 500 μg/24 hours) in acute organophosphate poisoning can be used without causing frequent clinical problems in resource-poor settings. Together with the extensive previous data on the safety of clonidine, our study suggests that clonidine has an acceptable safety profile at these doses when used in this setting. Reassuring in terms of safety, clinical outcomes in the lower dose groups were also (non-significantly) better than in the control group and in historical controls. These safety data should be interpreted with respect to the current high mortality from acute OP poisoning with standard treatment and the favorable data from animal studies on clonidine. We believe the data provide a basis to support further larger trials of clonidine to evaluate efficacy and gather further safety data.

## Abbreviations

OP: Organophosphorus (pesticide); WHO: World Health Organization; CNS: central nervous system; AChE: Acetylcholinesterase; MAP: mean arterial pressure; AV: atrio-ventricular; IQR: Interquartile range; BP: blood pressure; GCS: Glasgow coma score; SD: standard deviation; SEM: standard error of the mean; ANOVA: analysis of variance; RCT: randomised clinical trial; SACTRC: South Asian Clinical toxicology Research Collaboration

## Competing interests

The authors declare that they have no competing interests.

## Authors' contributions

All authors contributed extensively to the work presented in this paper. PMSP supervised the prospective study in the peripheral hospitals in Sri Lanka, did the data extraction, analysis and wrote the first draft of the paper. SFJ, RH, CA, and HK supervised the trial in their respective hospitals. NB designed the prospective study, provided statistical expertise, contributed to the analysis and interpretation of the data. AHD designed the prospective study and contributed to the analysis and the final draft. All authors reviewed and edited the final version of the manuscript.
